# Septicemia and pneumonia due to *Mycobacterium fortuitum* infection in a patient with extronodal NK/T-cell lymphoma, nasal type: A case report

**DOI:** 10.1097/MD.0000000000006800

**Published:** 2017-05-05

**Authors:** Jia Cong, Chenxi Wang, Li Ma, Shaoya Zhang, Jingwen Wang

**Affiliations:** a Department of Hematology, Beijing Tongren Hospital, Capital Medical University; b Department of Medical Oncology, Beijing Chest Hospital, Capital Medical University; c Department of Laboratory Medicine, Beijing Tongren Hospital, Capital Medical University, Beijing, China.

**Keywords:** lymphoma, *Mycobacterium fortuitum*, nontuberculous mycobacteria, pneumonia, septicemia

## Abstract

**Rationale:**

*Mycobacterium fortuitum* (*M.fortuitum*) is one of the rapidly growing nontuberculous mycobacterium (NTM) that is widespread in the environment. *M.fortuitum* can cause different types of disease including pulmonary disease, lymphadenitis, cutaneous disease, and disseminated disease. However, the infection presenting as septicemia is exceedingly rare.

**Patient concerns:**

A 48-year-old immunocompromised male with extranodal NK/T-cell lymphoma, nasal type was admitted to the hospital because of high fever for 10 days.

**Diagnoses:**

The pathogen identified twice in the hemoculture was *M.fortuitum,* but not in the sputum culture. The chest computed tomographic (CT) scan showed a chronic inflammatory infection.

**Interventions:**

The patient was treated with sulfamethoxazole and levofloxacin.

**Outcomes:**

The symptom of patient disappeared after the treatment for one week, and near-total absorption of the consolidation in CT after the treatment for one month. He continued the treatment for one year until the last negative hemoculture.

**Lessons:**

Although the *M.fortuitum* infection presenting as septicemia is rare, a high suspicion of *M.fortuitum* is required, particularly in the immunosuppressive patients. Timely and adequate treatment is necessary.

## 1 Introduction

*Mycobacterium fortuitum* is a ubiquitous environmental organism that has been isolated from soil, water, and other various sources.^[1]^ Although it is an infrequent pathogen, the incidence of *M fortuitum* infection are increasing with the wide application of immunosuppressants.^[2]^ The major clinical manifestations caused by *M fortuitum* present as pulmonary disease, lymphadenitis, cutaneous disease, and disseminated disease.^[2]^ The septicemia caused by *M fortuitum* is rare. Moreover, the clinical manifestation and pathologic feature of *M fortuitum* infection presenting as septicemia have not been well documented before since the low morbidity. Here, we first report a case with *M fortuitum* infection which causes septicemia and lung infection in an extranodal NK/T-cell lymphoma patient.

## 2 Case presentation

A 48-year-old man with extranodal NK/T-cell lymphoma was admitted with high fever, fatigue, and expectoration for a duration of 10 days. In February 2014, the patient was diagnosed as having extranodal NK/T-cell lymphoma, nasal type by tissue biopsy. The Ann Arbor stage of the patient was stage IIB, and international prognostic index score was 1. Afterwards, he received “sandwich” therapy with Gemcitabine, Oxaliplatin, and l-asparaginase chemotherapy for 5 cycles (q21d) and radiotherapy. The efficiency evaluation was partial response. After 1 month when he finished these therapies, the patient had marked fever, chills, and the highest temperature was 39°C without any apparent reason. Few hours later, the patient had back pain and sputum, and he felt weak, nausea, and anorexia. He was admitted to our hospital. There was a history of hepatitis B virus infection at his first admission.

The physical examination revealed the following: temperature 38.4°C; pulse: 94 beats/min and regular; respiratory rate: 21 breaths/min; blood pressure: 100/60 mmHg. The head, eyes, ears, nose, and throat examination was unremarkable. Superficial lymph nodes were touched without any enlargement. An examination of lungs revealed clear breath sounds. There was no clubbing, cyanosis, and edema. Physical examinations of the other organs were normal.

**Figure 1:**
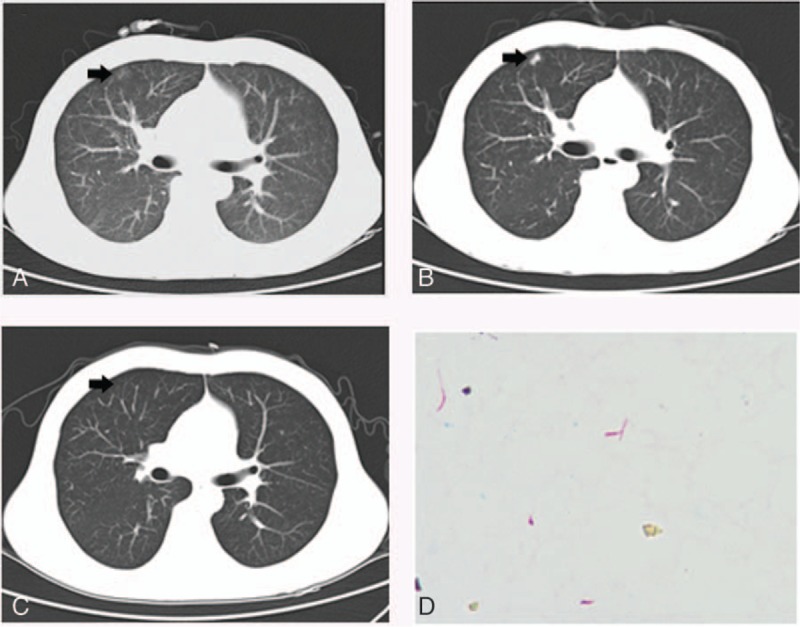
(A) A chest computed tomography (CT) scan showed cord and patch shadow with clear border on both sides. (B) A repeat chest CT scan indicated the lung lobe was more severe than admission. (C) A chest CT scan indicated that the consolidation disappeared. (D) The result of blood culture was *M fortuitum*. The acid-fast stain was positive.

Initial laboratory results included a normal leukocyte count of 4.82 × 10^9^ cells/L and hemoglobin 97 g/L. The coagulation function and platelet count were normal. C-reactive protein level was 87 μg/mL (reference range in our laboratory: 0–8 μg/mL). Procalcitonin (PCT) was negative. Initial electrolytes revealed the serum sodium was slightly decreased, and renal function and urine analysis were normal. Lanine aminotransferase: 63 U/L, aspartate transaminase: 73 U/L, lactate dehydrogenase: 235 U/L. His resting oxygen saturation was 97% with room air. His sputum examination showed *Candida tropicalis* 70%. Furthermore, the acid-fast staining of his sputum was negative. A chest computed tomographic (CT) scan showed cord and patch shadow with clear border on lung fields (Fig. 1A). His nasopharyngeal magnetic resonance imaging displayed that the lymphoma was no sign of progression.

He was placed on moxifloxacin 400 mg QD, vancomycin 1 g Q 12 h, and meropenem 1 g Q8 h empirically for 8 days. On the fourth day, his temperature descended to normal. After antibiotic treatment for eight days, the repeat chest CT showed a patchy consolidation shadow was more severe than that at the time of admission in the anterior segment of the right upper lob (Fig. 1B).

His blood culture indicated a septicemia of *M fortuitum* twice in 2 specimens collected at different time points. The acid-fast stain was positive (Fig. 1D). When we received these laboratory results, his antibiotic regimens were switched with sulfamethoxazole 1.92 g Q8 h and levofloxacin 0.5 g QD. The symptoms were released after 1 week of treatment. His temperature was normal all along during his rest time in hospital. A repeat CT scan after the first month of therapy showed near-total absorption of the consolidation (Fig. 1C). His treatment was continued for 12 months until the last negative blood culture.

## 3 Discussion

*M fortuitum* are widespread in the environment; overt disease is uncommon because of their low virulence.^[3]^ The pathogenicity of such an organism depends on the opportunity for transmission and the susceptibility of the host. In patients who are undergoing immunosuppressive therapy, *M fortuitum* infection appears to be more frequent, although there are some cases reported on the immunocompetent host.^[2]^ In the case report, this patient was diagnosed with extranodal NK-T-cell lymphoma and received chemotherapy for several cycles. He was an immunodeficient host who was susceptible to this pathogen.

Most of the clinical manifestations of *M fortuitum* infection are cutaneous lesion, typically following trauma or clinical procedures, post-traumatic ulcers, and lung disease.^[2]^ There are some other diseases caused by *M fortuitum* such as peritoneal dialysis-associated peritonitis, septic arthritis, corneal keratitis, and so on.^[4]^ Our case is unique in the fact that the patient was infected with *M fortuitum* presenting as septicemia and lung infection simultaneously. *M fortuitum,* which was cultivated from 2 different specimens of the patient's blood, can definitely prove the septicemia.

The common radiographic findings with *M fortuitum* infection were the presence of reticulonodular opacities, cavitary lesion, consolidation, and lobar volume decrease.^[2]^ This research reported the low positive (156 patients were identified with positive cultures for *M fortuitum* from respiratory specimens, only 1 positive culture for *M fortuitum* in sputum specimens).^[5]^ On the basis of his radiologic studies, we believed that the inflammation in his lung was related to *M fortuitum* infection. Although the patient's sputum culture for *M fortuitum* cannot show an evidence to support the diagnosis, his chest CT scan after taking sensitive antibiotics for *M fortuitum* showed improvement.

One pity of the case was that we failed to obtain the results of the drug sensitive testing about the patient's blood culture because of the artificial reason. What we use to control the *M fortuitum* infection was based on the related references: *M fortuitum* is usually susceptible to multiple oral antimicrobial agents, amikacin (100%), ciprofloxacin and ofloxacin (100%), sulfonamides (100%), imipenem (100%).^[4]^ Moreover, we did not get a positive sputum culture for *M fortuitum.* Therefore, we consider that the diagnosis of the disease relies on clinical, radiographic, bacteriologic evidences not only from sputum and tissues but also from blood.

In summary, septicemia and lung infection simultaneously from *M. fortuitum* are rare. A high suspicion of *M.fortuitum* is required, particularly in the immunosuppressive patients. The culture of the pathogens from the patient's body may be required to confirm the diagnosis. Advanced bacteriologic studies should be performed to help select appropriate treatment.

## 4 Consent

Written informed consent was obtained from the patient for publication of this case report and any accompanying images. A copy of the written consent is available for review by the Editor-in-Chief of this journal.
